# A software pipeline for processing and identification of fungal *ITS *sequences

**DOI:** 10.1186/1751-0473-4-1

**Published:** 2009-01-15

**Authors:** R Henrik Nilsson, Gunilla Bok, Martin Ryberg, Erik Kristiansson, Nils Hallenberg

**Affiliations:** 1 University of Gothenburg, Department of Plant and Environmental Sciences, Box 461, 405 30 Göteborg, Sweden; 2 The Sahlgrenska Academy at the University of Gothenburg, Department of Neuroscience and Physiology, Box 434, 405 30 Göteborg, Sweden

## Abstract

**Background:**

Fungi from environmental samples are typically identified to species level through DNA sequencing of the nuclear ribosomal internal transcribed spacer (*ITS*) region for use in BLAST-based similarity searches in the International Nucleotide Sequence Databases. These searches are time-consuming and regularly require a significant amount of manual intervention and complementary analyses. We here present software – in the form of an identification pipeline for large sets of fungal *ITS *sequences – developed to automate the BLAST process and several additional analysis steps. The performance of the pipeline was evaluated on a dataset of 350 *ITS *sequences from fungi growing as epiphytes on building material.

**Results:**

The pipeline was written in Perl and uses a local installation of NCBI-BLAST for the similarity searches of the query sequences. The variable subregion *ITS2 *of the *ITS *region is extracted from the sequences and used for additional searches of higher sensitivity. Multiple alignments of each query sequence and its closest matches are computed, and query sequences sharing at least 50% of their best matches are clustered to facilitate the evaluation of hypothetically conspecific groups. The pipeline proved to speed up the processing, as well as enhance the resolution, of the evaluation dataset considerably, and the fungi were found to belong chiefly to the *Ascomycota*, with *Penicillium *and *Aspergillus *as the two most common genera. The *ITS2 *was found to indicate a different taxonomic affiliation than did the complete *ITS *region for 10% of the query sequences, though this figure is likely to vary with the taxonomic scope of the query sequences.

**Conclusion:**

The present software readily assigns large sets of fungal query sequences to their respective best matches in the international sequence databases and places them in a larger biological context. The output is highly structured to be easy to process, although it still needs to be inspected and possibly corrected for the impact of the incomplete and sometimes erroneously annotated fungal entries in these databases. The open source pipeline is available for UNIX-type platforms, and updated releases of the target database are made available biweekly. The pipeline is easily modified to operate on other molecular regions and organism groups.

## Background

The fungal kingdom is thought to comprise upwards of 1.5 million species, a figure that contrasts sharply with the 97,000 species of fungi described so far [1,2]. The discrepancy between the estimated and the known fungal diversity is largely ascribable to the non-conspicuous nature of fungal life; most species are readily visible, and thus amenable for collection, only when they form fruiting-bodies or other propagation structures [3,4]. Yet fungi are ubiquitously present in all biota as mycelia or other, non-hyphal life stages, and studies have revealed that fungi may account for as much as 90% of the living biomass of some ecosystems [5]. The important ecological roles played by fungi in terms of nutrient recycling, symbiosis, and parasitism call for a deeper, taxonomy-oriented understanding of the mycoflora of the world, and one far more detailed than any fruiting-body based field inventory could ever bring about [6,7]. Mycologists of today thus face the daunting task of locating and identifying large sets of fungi from non-trivial substrates such as soil, decaying wood, and plant debris, often in the complete absence of any physical manifestation of the fungi present.

The last ten years have seen a surge in the use of DNA sequences as a means to seek to identify fungi from various substrates and in different life stages to species level [8,9]. The most frequently sequenced region for such purposes is the internal transcribed spacer (*ITS*) region of the nuclear ribosomal DNA [10]. Though it does not come without complications [11], this ~550 base-pair (bp), transcribed but non-coding region is present in upwards of 250 copies per cell [12], which makes it relatively straightforward to amplify even from scanty input material. The high level of variability in two of its three subregions (*ITS1 *and *ITS2*) coupled with the very low variability of the intercalary third subregion (*5.8S*) make the region an advantageous choice for assignment of a query sequence to species level or a higher fungal taxon [13,14]. Such inferences are typically achieved through similarity searches using BLAST [15] against the International Nucleotide Sequence Databases (INSD: GenBank, EMBL, DDBJ; [16]).

While BLAST is a powerful and versatile software package, its use over the web is dependent on server load and web traffic intensity. To compile and interpret its output in the case of multiple query sequences tends to be time-consuming and involve a significant amount of manual intervention. It is furthermore often necessary to re-run a certain proportion of the query sequences after the removal of overly conserved sequence segments – here the nuclear small subunit (*nSSU*/*16S*/*18S*), the *5.8S*, and the nuclear large subunit (*nLSU*/*25S*/*28S*) – in order to obtain fine-grained detail [13,17-19]. It may even be necessary to compile a multiple alignment of certain query sequences and their respective best matches and analyse the alignment under more powerful optimality criteria than similarity – such as phylogenetic analysis – in order to obtain sufficient resolution [cf. [20-23]]. To process an authentic set of fungal sequences obtained through environmental sampling thus often proves a lengthy and laborious process. The present study attempts a remedy in the form of a Perl-based software pipeline for rapid BLAST processing of large sets (10–100,000 s) of fungal query sequences. The pipeline streamlines functions such as automated *ITS2*-based BLAST runs for finer precision, the computation of multiple alignments for subsequent use, and the generation of comprehensible and easily analysed summaries of the results. The pipeline is designed to work in any UNIX-type environment (including MacOS X, Linux, and BSD) for which NCBI-BLAST, HMMER [24], and one or more of Clustal W [25], DIALIGN-TX [26], and MAFFT [27] are available.

To evaluate the performance of the pipeline on a large, authentic dataset, 350 *ITS *sequences were obtained from fungi growing on buildings in Sweden. Fungal growth on building material causes significant economic loss and is frequently connected to the Sick Building Syndrome [28]. The present sampling was carried out as a part of a larger study to characterize such fungal communities; detailed knowledge of the taxonomic affiliations of these fungi is likely to form a key element in assessing health risks caused by mass occurrence of fungi in moist damaged buildings [29].

## Implementation and methods

The pipeline [Additional file 1] was written in Perl 5 [30] and acts as a wrapper for NCBI-BLAST, HMMER, and Clustal W/DIALIGN-TX/MAFFT. By default, all fungal *ITS *sequences annotated as such in INSD are used as target database (approximately 110,000 sequences as of December 2008). Optionally, the user may choose to include only sequences identified to species level in the database. Tailored, biweekly updated versions of these databases are made available by the authors at [31].

For each query sequence, *blastn *of the NCBI-BLAST package is run locally, and the result is parsed to retrieve the 15 (default; adjustable) best (topmost) matches in INSD. An attempt is then made to locate and extract the *ITS2 *from the same query sequence using HMMER and the Markov models employed by Nilsson et al [14]. If successful, the *ITS2 *of the query sequence is used as the query in a new BLAST run against the same database, again with the 15 (default) best matches saved. The fifteen best matches of the first BLAST run are aligned jointly with the query sequence using Clustal W (default), DIALIGN-TX, or MAFFT to present the sequences in a more integrative context than the pairwise alignments of the BLAST output. The default value of the number of sequences to retain in the above steps was set to 15 as a product of the present focus on very similar sequences and the computational capacity at hand.

Upon completion of the BLAST runs, all query sequences that share at least 50% (default; adjustable) of their 15 best BLAST matches are clustered and aligned accordingly. Conspecific and closely related sequences are likely to be grouped into clusters in this step. Similarly, all sequences not a member of any such group are listed – in all likelihood, these singleton sequences represent species present only once among the query sequences. Ample screen output is given for each of the query sequences, and the results are summarized in a joint tab-separated file for viewing in any spreadsheet-type program (e.g., Open Office or Excel). The pipeline is released under the GNU-GPL software license version 2 and makes use of only freely available software. Although distributed over the web, the pipeline does not require an Internet connection to run.

The fungi were isolated from different kinds of building materials, e.g. wood, fibreboard, and linoleum mat, with varying extent of mould growth on the surface. The surface of the sample was scraped with a sterile needle and the scraped product was mixed with distilled water. The suspension was spread out on Petri dishes (Ø 9 cm) containing 2% malt extract agar (MEA) or dichloran 18% – glycerol agar (DG18). The samples were incubated in daylight at room temperature (20°C). Subsequently, fungal colonies were sub-cultured to new Petri dishes (Ø 6 cm) as part of the cleaning process. A total of 350 sequences were extracted using DNeasy Plant Mini Kit (QIAGEN, Hilden); amplified using READY-TO-GO PCR beads (QIAGEN) and the ITS1F and ITS4 primers; purified using the QiaQuick Spin Procedure Kit (QIAGEN); and sequenced using the ITS1F and ITS4 primers and the CEQ 2000XL DNA Analysis System (Beckman Coulter, Fullerton). Full details of the laboratory procedures are available in [32]. The sequences were pooled and fed to the pipeline using its default settings. Decisions of taxonomic affiliations were done considering the results from both the full *ITS *region and from the *ITS2 *region, including studying the alignments in non-trivial cases, with priority given to the *ITS2 *data where the results differed.

## Results and discussion

**Figure 1:**
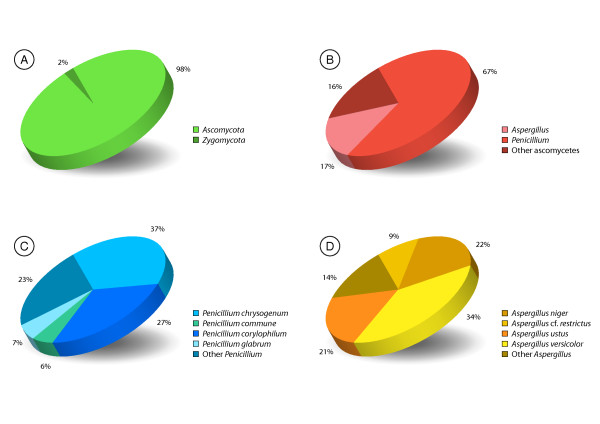
**Taxonomic distribution of the species recovered from the substrates**. The taxonomic distribution of the species in the pooled evaluation dataset over (A) the two fungal phyla retrieved, (B) the most common genera retrieved, (C) the most common species of *Penicillium*, and (D) the most common species of *Aspergillus*. The sequences were found to belong to 28 different fungal genera and 49 distinct species; an additional 11 sequences could not be assigned to species level with confidence due to the lack of fully identified reference sequences.

The 350 query sequences from mould growth on building material were found to be distributed among 42 distinct groups, each composed of mutually similar sequences to the exclusion of the other groups, and 27 singletons that did not match any other query sequence closely. The absolute majority (98%) of the fungal species recovered were found to belong to the *Ascomycota*, with a modest 2% of the sequences belonging to the *Zygomycota *(Fig. 1; Additional file 2). The two most common genera, *Penicillium *and *Aspergillus*, were totally dominant among the sequences with an occurrence of 58% and 16%, respectively. The most frequently occurring *Penicillium *species were *P. chrysogenum*, *P. corylophilum*, *P. glabrum*, and *P. commune*. The three latter species have as a rule not been identified to species level – but rather only to genus level – in prior biodiversity studies of building material due to the considerable difficulties in telling them apart using morphological analysis [33,34], a shortcoming that molecular data and the present software package are set to address. *Aspergillus versicolor*, *A. niger*, and *A. ustus *were the three most common species of the genus *Aspergillus*, which again represents a finer view than that presented in many surveys based on traditional techniques. Indeed, the view of the building mycoflora as revealed by the software pipeline shares many overall similarities with those from similar studies [35] but stands out through the level of resolution obtained on the species level [36,37]. The taxonomic and nomenclatural issues surrounding *Penicillium *and *Aspergillus *are far from trivial, however, and the lack of inclusive modern revisions and well-identified reference sequences are likely to have influenced the results of the present and many other efforts.

The evaluation dataset took two hours to run on a single-core 2.3 GHz Pentium 4 workstation. This is a considerable improvement in speed over any attempt at performing the corresponding BLAST runs over the web at INSD – particularly if complementary analyses have to be performed for some subset of the query sequences – and additional speed gains are to be attained if the pipeline is run on multiple-core processors. Manual inspection of the results revealed that the function to extract, and base the BLAST run on, the *ITS2 *gave a different taxonomic result than did the complete *ITS *region for about 10% of the cases (Additional file 2). This observation highlights the usefulness of excluding the very conserved genes encoding for the ribosomal small subunit, *5.8S*, and large subunit, particularly when sequences from species not present in the target database are used as query. The magnitude of the discrepancy between the taxonomic signal obtained from the *ITS2 *and from the entire *ITS *region (including any portions of the flanking genes) can be expected to vary among different groups of fungi.

The largest shortcoming of the present pipeline, as indeed with all sequence-based identification procedures, is the wanting state of taxonomic coverage and reliability in the public sequence databases. These vary among different taxonomic groups but form a tangible problem for the fungal entries. A mere 13,300 (14%) of the 97,000 described species of fungi are represented by at least one fully identified *ITS *sequence in INSD, a number that, in turn, pales in comparison with the estimated 1.5 million extant species of fungi. More than 10% of the fungal INSD sequences under study have, furthermore, been shown to be incorrectly identified to species level [38,39]. Adding to the complexity, 39% of the fungal *ITS *sequences in INSD are not identified to species level in the first place, and their sheer number often serves to mask the presence of explanatory, fully identified sequences in the BLAST hit list [40-42]. This is, however, a problem to which the present study offers a partial remedy in the form of the option to use only fully identified sequences in the BLAST reference database.

## Conclusion

We see the presented pipeline as a way to speed up the processing of large numbers of query sequences and to obtain extended functionality helpful for complementary, in-depth analyses. As with BLAST itself, however, the pipeline should be used on the understanding that definite assignment of a query sequence to species level is not amenable to algorithmic interpretation other than in a trivial sense, and in consideration of the fact that additional analysis steps may be needed, particularly for problematic query sequences.

## Availability and requirements

**Project name: **FungalITSPipeline

**Project home page: **

**Operating systems: **UNIX-type (MacOS X, Linux, UNIX/BSD)

**Programming language: **Perl

**Other requirements: **NCBI-BLAST, HMMER, optionally at least one of Clustal W, DIALIGN-TX, and MAFFT

**Licence: **GNU GPL 2

**Any restrictions to use by non-academics: **None other than those imposed by GNU GPL 2

**Notes: **The pipeline is available at the above address together with its documentation, the evaluation dataset, and biweekly updated BLAST database files. All these files are also bundled as additional data files with this manuscript. The pipeline is straightforward to adapt for other DNA regions and organism groups; new BLAST databases need to be assembled and the *ITS2 *mode should be turned off (or HMMs for the new gene need to be constructed).

## Competing interests

The authors declare that they have no competing interests.

## Authors' contributions

RHN wrote the chief part of the pipeline, to which MR and EK contributed with subroutines and suggestions. GB and NH undertook the sampling and DNA sequencing of the fungi of the evaluation dataset and were responsible for the study design. All authors contributed to the manuscript and approved its final version.

## Supplementary Material

Additional file 1 **The software pipeline.** The software pipeline described in the paper, together with its documentation, installation instructions, and the seed of a BLAST database. Click here for file

Additional file 2 **The evaluation dataset**. The evaluation dataset (in the FASTA format) and the results of its analysis (a summary in the OpenOffice/Excel spreadsheet format with multiple alignments in the ALN (Clustal W) format and a list of the species recovered in the PDF format). Click here for file
