# Supplementary material for: A software pipeline for processing and identification of fungal ITS sequences
Source: Source Code Biol Med. 2009 Jan 15;4:1. doi: 10.1186/1751-0473-4-1 (PMC2649129; doi:10.1186/1751-0473-4-1)
Supplement: Additional file 1 — The software pipeline. The software pipeline described in the paper, together with its documentation, installation instructions, and the seed of a BLAST database. [file 1751-0473-4-1-S1.zip › FungalITSPipeline/doc/manual.pdf]

User's guide

This document contains three sections:

1. Brief installation instructions (for those with some experience of UNIX-type environments)
2. Detailed installation instructions (for those with limited experience of UNIX-type environments)
3. Comments; Interpretation of the output

These instructions were written for MacOS X (particularly so for **section 2**). The Linux / BSD aficionado will probably find **section 1** to be the most relevant section for installation instructions.

1. Brief installation instructions (for those with some experience of UNIX-type environments)

- Make sure a recent version of NCBI-BLAST (http://www.ncbi.nlm.nih.gov/BLAST/download.shtml) is installed on your computer and can be reached through your path. Thus, when you open a Terminal and type blastall followed by enter, you should see output from BLAST.
- Make sure a recent version of the HMMER package (http://hmmer.janelia.org/#documentation) is installed on your computer and can be reached through your path. Thus, when you open a Terminal and type hmmpfam followed by enter, you should see output from HMMER.
- You will get the most out of the script if you have a multiple alignment program installed. The script comes prepared for all of Clustal W, MAFFT, and DIALIGN-TX, and it is recommended, but not required, that you install at least one of these. Clustal W (that is, the command line version of Clustal (ftp://ftp.ebi.ac.uk/pub/software/unix/clustalw/clustalw1.83.UNIX.tar.gz), not Clustal X, the graphical interface) compiles flawlessly on most platforms. Put it where your path reaches it. For the script to employ Clustal W (without any modifications to the former), Clustal W must start when you open a Terminal and type clustalw followed by enter. Note that the script has not been tested with the new Clustal W 2.0.
- The multiple alignment program MAFFT (http://align.bmr.kyushu-u.ac.jp/mafft/software/macosx.html) is optional as above. The script is tailored for the most advanced mode of MAFFT, linsi, so make sure it can be reached through your path (it will if you install MAFFT according to its instructions). Thus, when you open a Terminal and type linsi followed by enter, you should see output from MAFFT.
- The multiple alignment program DIALIGN-TX (http://dialign-tx.gobics.de/download) is optional as above. Install it according to its instructions such that it can be reached through your path; note however that you will have to specify the absolute location to the DIALIGN-TX configuration directory in the Perl script in order for DIALIGN-TX to work correctly.
- The zip archive http://andromeda.botany.gu.se/fungalpipeline.zip expands into a folder with all files needed to run the pipeline. Unzip it somewhere convenient. Enter the directory.
- The script comes bundled with all fungal ITS sequences in GenBank as of early August 2008. Updated versions of this file is released on a biweekly basis as http://andromeda.botany.gu.se/fungalpipelinedatabase.zip - unzip this file to the directory BLASTdatabase in the folder of the pipeline. Format the two files with formatdb of the BLAST package: formatdb -i fungalITSdatabase.fasta -p F and formatdb -i fungalITSdatabaseID.fasta -p F.
- The file indata/indata.fasta is where you place your query sequences. Use the FASTA format (see example below). It is advisable to stick with the generic FASTA format and to avoid, e.g., overly long or identical sequence names; the parser included is tolerant but there are obviously limits to what it can do. If you would just like to take the script for a spin, you could very well use the indata/indata.fasta that comes bundled with the script - that is, no further action needed. The bundled FASTA file contains the fungal sequences analysed in the paper presenting the

pipeline.

>Query1
CTTAGCTACGATACGCATCAGTACGTACGACTGACTAGACTGACTACGTACGACTACGTCAGACTGACTAC
GTACGACTGACTACGTACGTACGACTGACTACGTACG ...
>Query2
ACACGACTACTACCTAGCTAGCAGATGACACGACAGACGACGA
TGGATTAGCTGAGAGCCAGAGTAGAGACAGAGAGAGAGGATAT
...

- The default settings of the script include keeping the 15 best BLAST hits, using Clustal W for multiple alignment, and several other parameter values which are specified very early (line 24 and on) in the script. If you find these decisions satisfactory, you are ready to run the script. Do this through perl FungusPipeline.pl
- You will now see output on your screen. The output is tab separated and is echoed to a file; this means that the file is very easily interpreted but that the screen output will look rather messy (with columns out of place etc). This is OK; it is the output files we're primarily interested in. See section 3 for more information on the output files.

2. Detailed installation instructions (for those with limited experience of UNIX-type environments)

- In order to run this script, you will need to have sudo/superuser privileges ("root / Admin password") for your Mac (other UNIX). You will similarly have to install the Apple Xcode tools package (and, depending on your version of MacOS X, possibly also the x11 package) available on your MacOS X System CD/DVD in order to be able to compile source code. Please talk to your system administrator if you feel unsure about these steps. Note that they are mandatory and that you should not proceed unless these criteria are fulfilled.
- The below will use the directory /Users/henrik/temp/ as a temporary directory for various file operations. You will probably find that you don't have such a directory on your computer since it is user specific (and my user name is henrik). Open a Terminal, go to your home directory with cd ~ and create a folder called temp there with mkdir temp followed by enter. Enter the directory with cd temp followed by enter. Type pwd followed by enter. It will show you something like /Users/your_account/temp/. So when it says /Users/henrik/temp/ below, think /Users/your_account/temp/ instead.
- You will need to download a copy of NCBI-BLAST for local BLAST runs on your computer. In August 2008, I visited http://www.ncbi.nlm.nih.gov/BLAST/download.shtml and downloaded ftp://ftp.ncbi.nlm.nih.gov/blast/executables/LATEST/blast-2.2.18-universal-macosx.tar.gz to /Users/henrik/temp/
- Unpack the archive from a Terminal window: tar xvfz blast-2.2.18-universal-macosx.tar.gz and enter the new directory through typing cd blast-2.2.18 followed by enter (note that you might have to change the version number if you're doing this on a newer (or older) version of BLAST). We're only interested in the two binaries blastall and formatdb this time, so we copy them to where they can be reached through the path: sudo cp bin/blastall /usr/local/bin/ (sudo / root / Admin password required) followed by enter and sudo cp bin/formatdb /usr/local/bin/ followed by enter (or, if /usr/local/bin/ does not exist, use /usr/bin/ instead). blastall and formatdb are now installed on your computer; you can verify this by typing blastall and press enter - you should now see the blastall help message.
- To install the HMMER package, I went to http://hmmer.janelia.org/#documentation and downloaded ftp://selab.janelia.org/pub/software/hmmer/CURRENT/hmmer-2.3.2.tar.gz which I put in /Users/henrik/temp/ and unpacked: tar xvfz hmmer-2.3.2.tar.gz followed by enter.

- I enter the new directory with cd hmmer-2.3.2 and follow the instructions in the file INSTALL such that I type ./configure and press enter; then I type make and press enter; then I type make check and press enter; and finally I type sudo make install and press enter. The HMMER package should now have been compiled and installed on your computer; you can check this by typing hmmpfam and press enter - you should now see HMMER output. Note that you might have to change the above file names if you're installing another verion of HMMER than 2.3.2. This step (i.e., the compilation) requires that the Xcode tools / x11 packages on your MacOS X System CD/DVD are installed. [On one of the many Macs I tried this on, the sudo make install step failed for some obscure reason. As long as the preceding steps worked fine, this should be no problem, though you will have to copy the hmmpfam binary to /usr/local/bin/ (or /usr/bin/ as above) by hand: sudo cp src/hmmpfam /usr/local/bin/ followed by enter.]
- It is optional but recommended to let the script oversee multiple alignment of the query sequences jointly with their respective best BLAST matches. The following steps describes how to install Clustal W (that is, the command-line tool, not Clustal X, the graphical interface version), DIALIGN-TX, and MAFFT. To install Clustal W, I went to http://www.bioinformatics-toolkit.org/Help/Topics/clustalw.html and downloaded the file ftp://ftp.ebi.ac.uk/pub/software/unix/clustalw/clustalw1.83.UNIX.tar.gz to /Users/henrik/temp/ where I unpacked it: tar xvfz clustalw1.83.UNIX.tar.gz followed by enter.
- I enter the new directory with cd clustalw1.83 followed by enter and simply type make followed by enter. I copy the resulting clustalw binary to /usr/local/bin/ (or /usr/bin/ as above) with sudo cp clustalw /usr/local/bin/ . Clustal W is now ready to run - when you type clustalw followed by enter in a Terminal, you should see it start. Note that the script remains untested with the new Clustal W 2.0.
- To install MAFFT, I went to http://align.bmr.kyushu-u.ac.jp/mafft/ and download http://align.bmr.kyushu-u.ac.jp/mafft/software/mafft-6.603-without-extensions-src.tgz to /Users/henrik/temp/ where I unpacked it: tar xvfz mafft-6.603-without-extensions-src.tgz followed by enter (the MAFFT development team is very productive, and you'll probably see a different version number than the one above when you get there. If so, please modify the relevant file names accordingly). I enter the new directory with cd mafft-6.603-without-extensions followed by enter and follow the instructions in the file readme: I type cd core followed by enter; I type make clean followed by enter; I type make followed by enter; I type sudo make install followed by enter; and finally I type cd .. followed by enter. MAFFT should now be installed; if you type linsi followed by enter in a Terminal, you should see MAFFT output.
- DIALIGN-TX is something of a special case. You should install it in a permanent folder which you do not plan to move anywhere (since you need to specify the location of the DIALIGN-TX configuration directory every time you run it). To install DIALIGN-TX I go to http://dialign-tx.gobics.de/download and download the file http://dialign-tx.gobics.de/DIALIGN-TX_1.0.1.tar.gz to /Users/henrik/program/DNA/alingment/ where I unpack it with tar xvfz DIALIGN-TX_1.0.1.tar.gz followed by enter. I enter the new directory with cd DIALIGN-TX_1.0.1 followed by enter and type ./install.sh followed by enter. I then copy the new binary bin/dialign-tx to /usr/local/bin/ (or /usr/bin/ as above) with sudo cp bin/dialign-tx /usr/local/bin/ followed by enter. You will need to add the location of the configuration directory of DIALIGN-TX to the fungal pipeline (described below). For now, let's just note the absolute path to this directory: /Users/henrik/program/DNA/alingment/DIALIGN-TX_1.0.1/conf/ - make sure you take down a note of yours (the path to the DIALIGN-TX directory with "/conf/" appended to it).
- The zip archive http://andromeda.botany.gu.se/fungalpipeline.zip contains the fungal pipeline. Download it to, say, /Users/henrik/program/ and extract it with unzip fungalpipeline.zip followed by enter. A folder called FungalPipeline will be created. Enter it with cd

/Users/henrik/program/FungalPipeline followed by enter.

- The file indata/indata.fasta is where you place your query sequences. Use the FASTA format (see example below). It is advisable to stick with the generic FASTA format and to avoid, e.g., overly long or identical sequence names; the parser included is tolerant but there are obviously limits to what it can do. If you would just like to take the script for a spin, you could very well use the indata/indata.fasta that comes bundled with the script - that is, no further action needed. The bundled FASTA file contains the fungal sequences analysed in the paper presenting the pipeline.

```
>Query1
CTTAGCTACGATACGCATCAGTACGTACGACTGACTAGACTGACTACGTACGACTACGTCAGACTGACTAC
GTACGACTGACTACGTACGTACGACTGACTACGTACG ...
>Query2
ACACGACTACTACCTAGCTAGCAGATGACACGACAGACGACGA
TGGATTAGCTGAGAGCCAGAGTAGAGACAGAGAGAGAGGATAT
...
```

- The default settings of the script include keeping the 15 best BLAST hits, using Clustal W for multiple alignment, and several other parameter values which are specified very early (line 24 and on) in the script. If you find these decisions acceptable, you are ready to run the script. Do this through perl FungusPipeline.pl
- You will now see output on your screen. The output is tab separated and is echoed to a file; this means that the file is very easily interpreted but that the screen output will look rather messy (with columns out of place etc). This is OK; it is the output files we're interested in. See section 3 for more information on the output files.
- To analyse your own sequences, open the file indata/indata.fasta in your favourite text editor and replace the sequences in it with your own. Make sure to use the FASTA format (see example above). Save the file back and run the pipeline as describe above.
- The script comes bundled with all fungal ITS sequences in GenBank as of early August 2008 as reference sequences (that is, the sequences to which BLAST compares your sequences). Updated versions of this file is released on a biweekly basis as http://andromeda.botany.gu.se/fungalpipelinedatabase.zip - download and unzip this file (unzip fungalpipelinedatabase.zip followed by enter) to the directory BLASTdatabase in the folder of the pipeline (/Users/henrik/program/FungalPipeline/ in the example above). Format the two files with formatdb of the BLAST package: formatdb -i fungalITSdatabase.fasta -p F followed by enter and formatdb -i fungalITSdatabaseID.fasta -p F followed by enter. You will now be up to date.
- DIALIGN-TX: If you plan on using DIALIGN-TX, you must open the script FungalPipeline.pl in your favourite text editor and change the line my $dialigntxconfdir="/home/henrik/temp/DIALIGN-TX_1.0.1/conf/"; to point to the configuration directory of DIALIGN-TX as installed on your computer (see above for comment). In my case that is /Users/henrik/program/DNA/alingment/DIALIGN-TX_1.0.1/conf/

3. Interpretation of the output

- A unique output directory will be created for each run in the directory outdata. This unique directory will carry the name of the date (with an appended number: 1 for the first run that day, 2 for the second etc). So for the first run you did on August 10, 2008 you will find the output files in outdata/2008-8-10-1/.
- In this particular case, the summary of the run is saved as outdata/2008-8-10-1/outdata.csv. This is a tab-separated file which you can open in, e.g., Excel or Open Office (the spreadsheet program might prompt you to specify that tabs are used to delimit the data units of the file). The upper

part of the file lists, in order of appearance in the input file, your query sequences and their 15 (default) best matches along with the length of the query sequence and the BLAST scores and E-values. The left set of columns pertains to the results of the BLAST run of the entire query sequence whereas the right set of columns pertains to the results of the BLAST run of the ITS2 region of the query sequence. Not all query sequences will have this right set of columns since it is contingent on whether the ITS2 was extracted from the query sequence or not. The ITS2 region is highly variable, which makes very useful for species identification attempts (be it that it may not be variable enough for all taxa of fungi). In comparison, the whole of the ITS region might present some problems when significant portions of the juxtaposed nSSU and nLSU (and even 5.8S) genes are present in the query sequence [and the species in question is not present in the target dataset]. Since these genes are comparatively conserved across large sections of the fungal kingdom, they are likely to nearly always find good matches in the target database. Thus, a good match to say 150 bp of nSSU will bring such target sequences up in the BLAST hit list, even if the juxtaposed ITS1 does not produce a high-quality match to the query sequence; this might lure the unaware user to believe that the match is better than it really is. The 5.8S has a similar effect since it is even more conserved than nSSU and nLSU. In extracting the ITS2 and BLAST:ing it in isolation, the ITS2 mode of the fungal pipeline does its part to sidestep these potential problems. Then again, there is a lot of valuable information in the whole-ITS BLAST process as well. So please consider both the results from the complete ITS region and from the ITS2 region (they are shown side by side in the output) when you interpret your BLAST results.

- The lower part of the file contains two lists: a list of clusters and a list of singletons. A cluster is defined as all sequences that shared at least 50% (default) of their 15 (default) best BLAST matches. Thus, all sequences in a cluster can be suspected to be very closely related. We perceive the clustering step as a way of not having to go through the output for each and every one of potentially thousands of query sequences but rather to examine one sequence per cluster to get an idea of the taxonomic affiliation of the cluster. That said, please be aware that any two sequences in a cluster may share 50% of their hits even if these are the 10 first hits for one of the sequence and the 10 last for the other such that their conspecificity does not need to be certain. A singleton is a sequence that was found not to share at least 50% of the 15 (default) best BLAST matches with any other sequence. We perceive these as taxa present only once in the indata. The clusters were restricted so that any one query sequence can only belong in one cluster - this can be changed in the script. The clustering step is based on the outcome of the BLAST results of the whole query sequence (since not all sequences have an ITS2, which would lead to incomplete clustering where not all sequences were either present in a cluster or come out as a singleton). The cut-off percentage is set through the parameter $threshold (default: $threshold=0.5). The default value of 50 % is obviously somewhat arbitrary, but it is still rather strict in terms of BLAST hit lists. Quite few sequences (even conspecific ones) share 75 % of the top 15 matches (the better represented a species is, the more identical (give or take) hits you will get, and the less likely that two different sequences will bring up the very same accession numbers due to the way BLAST orders its hit lists). Similarly, if you reduce the parameter to 30 %, you will get larger, more relaxed clusters. Our suggestion is that you start with 50 % and see what makes the most sense for your taxa. A value of 50% was found to perform well on our data.
- If you chose to compute alignments during the run, the directory outdata/2008-8-10-1/alignments will contain one alignment for every query sequence (featuring the query sequence aligned to its 15 (default) best BLAST hits). It similarly contains one alignment for every cluster; these alignments feature all query sequences found to belong to the clusters in question. Please keep in

mind that viewing the underlying alignments gives you a much better view of relatedness than BLAST scores and E-values do. We perceive these as very important to the interpretation process.

- The default number of sequences to retain is 15 (see above). We arrived at this value through BLAST:ing 100 random fungal ITS sequences and noting how many very similar matches they had. The value of 15 is slightly more than that average, which serves our purpose as it will typically give you perhaps 10 highly similar sequences and 5 slightly less similar sequences (with respect to the query sequence at hand). In a way, that gives you both an ingroup and an outgroup. You should probably set this value to be the same as the number of matches you normally look at in a BLAST hit list. Note, however, that high values will increase the computational burden (if alignments are computed, anyway). Also for the reason that the value will have an impact on the nature of the clusters, you should probably strive to keep this number reasonably low. We'd be happy to learn of your experiences with this parameter and how different values perform on different datasets.
- To modify the script to work on other genes and organism groups is pretty straightforward but may require some knowledge in Perl depending on how far you would like to go. Note how the FASTA files that underpin the BLAST databases are constructed (specifically how the entries are named) and assemble a similar FASTA file for your organism group / gene. Format it as a nucleotide database using `formatdb` of the BLAST package (see above). Unless you want something similar to the ITS2 mode for the new gene/organism group, you're good to go. Otherwise you'll need to compile new HMMs (using `hmmpfam` of the HMMER package), try them out on your sequences to estimate cut-off values for where you're starting to see false matches, and implement these values in the script. Copy the HMMs to the HMMs directory and change the file names / search paths as needed in the script. That should be it.
