# Supplementary material for: A software pipeline for processing and identification of fungal ITS sequences
Source: Source Code Biol Med. 2009 Jan 15;4:1. doi: 10.1186/1751-0473-4-1 (PMC2649129; doi:10.1186/1751-0473-4-1)
Supplement: Additional file 2 — The evaluation dataset. The evaluation dataset (in the FASTA format) and the results of its analysis (a summary in the OpenOffice/Excel spreadsheet format with multiple alignments in the ALN (Clustal W) format and a list of the species recovered in the PDF format). [file 1751-0473-4-1-S2.zip › AdditionalFile2/SpeciesSummary.pdf]

Species recovered	Number of occurrences
Penicillium chrysogenum	88
Penicillium corylophilum	61
Aspergillus versicolor	20
Penicillium brevicompactum	16
Penicillium glabrum	16
Aspergillus niger	13
Penicillium commune	13
Aspergillus ustus	12
Penicillium citreonigrum	7
Sydowia polyspora	7
Penicillium biourgeianum	6
Aspergillus cf. *sestrictus*	5
Penicillium cf. *islandicum*	5
Penicillium roseopurpureum	5
Penicillium sumatraense	4
Aspergillus rubrum	3
Emericella nidulans	3
Exophiala xenobiotica	3
Lecythophora hoffmannii	3
Oidiodendron griseum	3
Penicillium olsonii	3
Rhizomucor variabilis	3
Alternaria alternata	2
Aspergillus sydowii	2
Cephalotheca cf. *purpurea*	2
Chaetomium sp.	2
Crivellia papaveracea	2
Eurotium amstelodami	2
Gibberella fujikuroi	2
Gibberella moniliforme	2
Hypocrea lixii	2
Mucor plumbeus	2
Penicillium aurantiovirens, cyclopium	2
Ramichloridium anceps	2
Simplicillium lanosiniveum	2
Ulocladium multiforme	2
cf. *Arachnomyces*	1
Aspergillus amstelodami, hollandicus	1
Chaetomium globosum	1
Cladosporium sphaerospermum	1
Cladosporium variabile	1
Engyodontium album	1
Epicoccum nigrum	1
Fusarium sp.	1
Hypocrea viridescens	1
Kabatiella / Aurebasidium	1
Oidiodendron griseum	1
Paecilomyces variotii	1
Penicillium coffeae	1
Penicillium cf. *commune*	1

Penicillium crustosum	1
Penicillium dangeardii	1
Penicillium decumbens	1
Penicillium granulatum	1
Penicillium cf. *raistrickii*	1
Penicillium sclerotigenum	1
Phoma glomerata	1
Phoma herbarum	1
Stachybotrys chartarum	1
